# Study protocol of a randomized, double-blind, placebo-controlled, multi-center trial to treat antipsychotic-induced weight gain: the Metformin-Lifestyle in antipsychotic users (MELIA) trial

**DOI:** 10.1186/s12888-020-02992-4

**Published:** 2021-01-05

**Authors:** Nini de Boer, Sinan Guloksuz, Caroline van Baal, Leonie Willebrands, Jeroen Deenik, Christiaan H. Vinkers, Inge Winter-van Rossum, Janneke Zinkstok, Ingeborg Wilting, Jasper B. Zantvoord, Frank Backx, Wilma E. Swildens, Marieke Schouw, Jan Bogers, Folkwin Hulshof, Rudolf de Knijff, Peter Duindam, Mike Veereschild, Maarten Bak, Geert Frederix, Lieuwe de Haan, Jim van Os, Wiepke Cahn, Jurjen J. Luykx

**Affiliations:** 1 Department of Psychiatry, UMC Utrecht Brain Center, University Medical Center Utrecht, Utrecht University, HP A01.126, P.O. Box 85500, 3508 Utrecht, GA The Netherlands; 2 Department of Psychiatry and Neuropsychology, School for Mental Health and Neuroscience, Maastricht University Medical Centre, Maastricht, The Netherlands; 3 Department of Psychiatry, Yale University School of Medicine, New Haven, CT USA; 4 Department of Biostatistics and Research Support, Julius Center, University Medical Center Utrecht, Utrecht University, Utrecht, The Netherlands; 5 GGz Centraal Mental Health, Amersfoort, The Netherlands; 6 Department of Psychiatry and Department of Anatomy and Neuroscience, Amsterdam University Medical Center, University of Amsterdam, Amsterdam, The Netherlands; 7 GGZinGeest Mental Health, Amsterdam, The Netherlands; 8 Department of Clinical Pharmacy, University Medical Center Utrecht, Utrecht University, Utrecht, The Netherlands; 9 Department of Rehabilitation, Physiotherapy Science & Sport, University Medical Center Utrecht, Utrecht University, Utrecht, The Netherlands; 10 Altrecht Mental Health Care Institute, Utrecht, The Netherlands; 11 Inholland University of Applied Sciences, Interprofessional Mental Health Care, department Nursing, Amsterdam, The Netherlands; 12 GGZ Rivierduinen, Oegstgeest, The Netherlands; 13 GGNet Mental Health, Warnsveld, The Netherlands; 14 Mondriaan Mental Health, Maastricht, The Netherlands; 15 Department of Public Health, Julius Center, University Medical Center Utrecht, Utrecht University, Utrecht, The Netherlands; 16 Arkin GGZ, Amsterdam, The Netherlands; 17 Department of Psychosis Studies, Institute of Psychiatry, Psychology & Neuroscience, King’s College London, London, UK; 18 Department of Translational Neuroscience, UMC Utrecht Brain Center, University Medical Center Utrecht, Utrecht University, Utrecht, The Netherlands

**Keywords:** Antipsychotic-induced weight gain (AiWG), Schizophrenia, Metformin, Lifestyle

## Abstract

**Background:**

Antipsychotic-induced Weight Gain (AiWG) is a debilitating and common adverse effect of antipsychotics. AiWG negatively impacts life expectancy, quality of life, treatment adherence, likelihood of developing type-2 diabetes and readmission. Treatment of AiWG is currently challenging, and there is no consensus on the optimal management strategy. In this study, we aim to evaluate the use of metformin for the treatment of AiWG by comparing metformin with placebo in those receiving treatment as usual, which includes a lifestyle intervention.

**Methods:**

In this randomized, double-blind, multicenter, placebo-controlled, pragmatic trial with a follow-up of 52 weeks, we aim to include 256 overweight participants (Body Mass Index (BMI) > 25 kg/m^2^) of at least 16 years of age. Patients are eligible if they have been diagnosed with schizophrenia spectrum disorder and if they have been using an antipsychotic for at least three months. Participants will be randomized with a 1:1 allocation to placebo or metformin, and will be treated for a total of 26 weeks. Metformin will be started at 500 mg b.i.d. and escalated to 1000 mg b.i.d. 2 weeks thereafter (up to a maximum of 2000 mg daily). In addition, all participants will undergo a lifestyle intervention as part of the usual treatment consisting of a combination of an exercise program and dietary consultations. The primary outcome measure is difference in body weight as a continuous trait between the two arms from treatment inception until 26 weeks of treatment, compared to baseline. Secondary outcome measures include: 1) Any element of metabolic syndrome (MetS); 2) Response, defined as ≥5% body weight loss at 26 weeks relative to treatment inception; 3) Quality of life; 4) General mental and physical health; and 5) Cost-effectiveness. Finally, we aim to assess whether genetic liability to BMI and MetS may help estimate the amount of weight reduction following initiation of metformin treatment.

**Discussion:**

The pragmatic design of the current trial allows for a comparison of the efficacy and safety of metformin in combination with a lifestyle intervention in the treatment of AiWG, facilitating the development of guidelines on the interventions for this major health problem.

**Trial registration:**

This trial was registered in the Netherlands Trial Register (NTR) at https://www.trialregister.nl/trial/8440 as NTR NL8840 on March 8, 2020.

## Background

Antipsychotics are the mainstay treatment modality for schizophrenia. Antipsychotic-induced Weight Gain (AiWG) is a debilitating and prevalent adverse effect. Antipsychotics are associated with weight gain, lipid and glucose dysregulation and therefore increase the risk for the development of metabolic syndrome (MetS) [1–5]. After the initation of antipsychotic treatment in medication-naïve patients, the average increase in Body Mass Index (BMI) is 1.8 units in 12 weeks and 3.9 units in 48 weeks [6]. The relative risk for obesity is up to two in schizophrenia patients using antipsychotics compared to the general population [7]. The increased obesity rate in schizophrenia patients can be mainly attributed to the use of antipsychotics [8]. Obesity is a major health problem resulting in increased morbidity and mortality, thus decreasing life expectancy and increasing pressure on healthcare budgets [9, 10]. Schizophrenia patients have a reduced life expectancy of 20% compared to the general population and approximately one third of patients with schizophrenia have MetS [5, 11]. Moreover, the relative risk of MetS for people with severe mental illness is 1.58 compared to the general population [11]. Importantly, all antipsychotics are to variable degrees associated with AiWG. For example, in a retrospective study of many antipsychotics, AiWG was highest for olanzapine with 4.2 kg and lowest for amisulpride at 1.8kgs measured over a period of 3 years [12]. Aripiprazole and haloperidol have been reported to result in relatively mild or in some studies no AiWG. AiWG also negatively impacts quality of life, treatment adherence, likelihood of developing type-2 diabetes and readmission rate [13–15]. Considering the growing mortality gap between schizophrenia patients and the general population, additional management strategies to increase the life expectancy of schizophrenia patients are necessary [16].

Despite these broad clinical implications of AiWG, treatment of AiWG has not yet been optimized. Most guidelines, including the Dutch national guideline for schizophrenia, recommend the following interventions for AiWG: 1) switching of antipsychotic; or 2) weight reduction through lifestyle interventions (diet and exercise) [17]. The impact of switching antipsychotic treatment is limited as the vast majority of antipsychotics is associated with weight gain [18]. Weight reduction through lifestyle interventions is viable but known for its relatively variable efficacy. For example, a recent study found a statistically significant effect of lifestyle interventions on weight in people with severe mental illness that was considered clinically insignificant, while a meta-review of meta-analyses found individual lifestyle counselling and exercise alone (i.e. planned, structured intensive physical activity) as the most effective weight reduction intervention in patients with schizophrenia [19, 20]. Of note, integrated interventions combining multiple lifestyle components (e.g. exercise and diet) delivered by qualified professionals (e.g. dietitian, exercise professionals) may be most effective for health-related outcomes and adherence, although such interventions have been less studied yet [21–23]. In this context, many guidelines, including the 2018 WHO guidelines and the Dutch national guideline for schizophrenia, recommend lifestyle interventions as first-line strategy for the management of physical health in people with severe mental illness, including AiWG [17, 24]. However, given the extensive challenge to clinically significantly reduce AiWG and the only partial contribution of lifestyle interventions to help lose weight, there is a clear need for additional (combined) strategies to manage AiWG.

Metformin is a promising agent in the treatment of AiWG. Metformin generally promotes satiety and increases Glucagon-like Peptide (GLP-1), thus often resulting in reduced energy intake [25]. Clozapine reduces the production of GLP-1 and therefore is a viable treatment option for clozapine-induced weight gain [25]. Meta-analyses conclude that of all agents studied as monotherapy for AiWG, metformin is most effective in attaining weight loss for child and adolescent schizophrenia patients and for those patients who use clozapine [26, 27].

Recently, a meta-analysis investigated the combination of metformin and lifestyle interventions as a treatment for AiWG, reporting only one double-blinded trial on this topic [28, 29]. Additionally, five open label studies were included, published in Chinese journals which are not indexed by Pubmed and found to be of poor quality [29–34]. That double-blinded, well powered randomized controlled trial of sufficient quality comparing lifestyle interventions with and without concomitant use of metformin in Chinese antipsychotic users found metformin, lifestyle interventions and the combination of metformin and lifestyle interventions to be superior to placebo [28]. The best results on body weight, waist circumference and BMI were obtained for the combination of lifestyle plus metformin. Important limitations of that trial and any previous trials of AiWG done with metformin are the strict inclusion criteria with regards to diagnosis (no other diagnoses than first-episode schizophrenia allowed in that previous trial) and the amount of AiWG, thus not adequately reflecting schizophrenia patients encountered in general practice [28]. Furthermore, a very extensive exercise program (seven times a week) was implemented in this trial and we question the applicability of such an intensive intervention. Moreover, the duration of treatment was short (12 weeks) and the doses of metformin were relatively low (mostly around 1000 mg daily), even though efficacy may be better with higher doses without a negative impact on tolerance [35, 36]. Finally, all aforementioned trials were conducted before the COVID-19 pandemic, which has heavily impacted both research and clinical activities across the globe. Particularly lifestyle sessions with many individuals, often placed in the same indoor space, are challenging to conduct in the current era with social distancing and lockdowns in place in several countries and the possible scenario of a future virus affecting global health.

## Objectives

In sum, the treatment of AiWG is currently very challenging and few interventions have been investigated in well powered trials of sufficient quality. Therefore, this randomized, double blind, multicenter, placebo-controlled, pragmatic trial aims to evaluate the effect of metformin doses up to 2000 mg daily for AiWG as an additional strategy next to treatment as usual including lifestyle interventions, by comparing metformine plus the lifestyle intervention with placebo plus the lifestyle intervention. In the event of a significantly greater reduction of AiWG following metformin use in combination with care as usual including a lifestyle intervention, guidelines can include metformin for AiWG. Furthermore, we aim to investigate the difference in metformin-associated reduction of AiWG between participants using clozapine versus other antipsychotics; between participants on high-AiWG risk agents (i.e. olanzapine, clozapine, quetiapine, risperidone and paliperidone) compared to all other antipsychotics; and the difference between self-reported weight gain due to antipsychotic use versus weight gain due to other reasons [2, 12]. At last, we aim to examine whether lifestyle interventions in combination with metformin or placebo improve metabolic traits, quality of life, general physical and psychological health, cost effectiveness, and whether genetic liability to BMI and MetS allows adequate estimation of weight reduction following initiation of metformin and lifestyle interventions.

Metformin and placebo both combined with treatment as usual including lifestyle interventions are the conditions we wish to evaluate with difference in body weight as the primary outcome measure. We hypothesize that metformin given adjunctively to lifestyle interventions will lead to a higher level of weight reduction and better quality of life, compared to placebo in combination with lifestyle interventions.

## Methods

### Study setting

This multi-center trial will be conducted at the University Medical Centre Utrecht (UMCU), Mondriaan Mental Health Maastricht and GGNet Mental Health in the Netherlands. The estimated duration of this study is 3.5 years (including a participants’ enrolment phase) and participants will be followed-up for 52 weeks. The study has been approved by the Medical Research Ethics Committee Utrecht and was registered at the Netherlands Trial Register (NL8840). The study will be conducted according to the principles of the Declaration of Helsinki (amended version in October 2013) and following Good Clinical Practice guidelines from the European Medicines Agency (ICH E6, R2) and the Medical Research Involving Human Subjects Act (WMO).

### Study population

One hundred tewnty-eight participants with a schizophrenia spectrum disorder will be followed-up for 26 weeks. With an estimated attrition rate of 50% based on attrition rates of previous trials targeting schizophrenia patients, a total of 256 participants will be recruited from the participating and referring centres in the Netherlands (see ‘sample size calculation’ below) [37]. Two groups of subjects with psychotic disorders will be recruited: 1) general patients suffering from psychosis who use a range of antipsychotics; and 2) those considered (relatively) treatment-resistant who are treated with clozapine. We specifically chose both patients on clozapine and patients on other antipsychotics given possible differences in the efficacy of metformin for both patient groups: metformin may be a particularly viable treatment modality for clozapine-induced weight gain as clozapine-induced decreases in GLP-1 may be partially offset by metformin’s GLP-1 increasing effects [25]. Eligibility of all possible participants will be determined following intake questions, physical examination and laboratory findings. Should any of the results from the intake visit require immediate attention, the treating physician will contact the participant’s general practitioner (GP).

### Recruitment

Participating and referring sites will be recruiting. At participating sites, participants will be recruited by treating psychiatrists, psychologists and general practitioners. The participants will be informed and asked for written informed consent by a research-physician or P.I. Referring sites provide contact details of possible eligible participants to participating sites after the patient has provided consent. In addition, referring sites will ask participants for their consent that a participating site can approach the participant. Referring sites have no part in the inclusion process. Referring and participating sites can use posters and brochures to recruit eligible participants. Furthermore, physician brochures will be used to inform treating physicians and to support the process of recruitment. Enrolment of participants, obtaining informed consent and randomization will be performed by authorized personnel of the research team of participating sites.

### Inclusion criteria

1. Diagnosis of schizophrenia spectrum disorders according to DSM-IV-TR or DSM-5 criteria as summarized in DSM-IV or DSM-5 chapter Schizophrenia Spectrum and other Psychotic Disorders, except for substance/medication-induced psychotic disorder, psychotic disorder due to another medical condition, catatonia associated with another mental disorder, catatonic disorder due to another medical condition, and unspecified catatonia;
2. Antipsychotic use for at least 3 months (as most AiWG occurs in the first weeks to months of treatment);
3. Willingness to participate in a lifestyle intervention;
4. Dutch speaking and reading;
5. Mentally competent according to the treating physician;
6. Able to give informed consent;
7. At least 16 years of age;
8. Overweight (BMI > 25).

### Exclusion criteria

1. Neurodegenerative extrapyramidal disease;
2. Metformin-related contraindications: i.e., conditions predisposing to tissue hypoxia (such as congestive heart failure, recent myocardial infarction and respiratory failure), metabolic acidosis, precoma diabeticum, kidney failure (glomerular filtration rate (GFR) < 30 ml/min) and conditions predisposing to kidney failure (e.g. dehydration, infections and hypovolemic shock), disorders in the use of alcohol defined as > two reported consumptions daily and/or a gamma-glutamyltransferase (GGT) of over 60 U/L, and liver failure;
3. Current use of medication(s) that inhibit(s) metabolism of metformin or otherwise interacts with metformin (in particular those with possible detrimental effects on kidney functioning):
  -Non-steroidal anti-inflammatory drugs (NSAIDs).
  -Angiotensin converting enzyme-inhibitors (ACE-inhibitors).
  -Angiotensin receptor blockers (ARBs).
  -Diuretics.
  -Organic cation transporters (OCT) -1 and 2 inhibitors (e.g. cimetidine, dolutegravir, isavuconazol, trimethoprim, vandetanib, crizotinibib, vandetanib, and verapamil) and inductors (e.g. rifampicin);
4. Vitamin B12 deficiency defined as a Vitamin B12 serum level of < 148 pmol/L [38];
5. Diabetes mellitus according to NHG-criteria [39];
6. Pregnant or breast feeding women or women of child bearing age using no contraceptives.

### Randomization and blinding

After eligibility screening and providing written informed consent, participants will be randomized using block-randomization with a 1:1 allocation to 1) placebo; or 2) metformin. Randomization will be stratified for clozapine use versus other antipsychotic use additional to stratification for participating sites using a computer-generated double-blind application developed by the UMC Utrecht’s Data Management department. The block sizes will not be disclosed. Allocation concealment will be facilitated by releasing the randomization code only after the participant has completed all baseline assessments and has been included in the trial.

The participant, caregiver, investigator and outcome assessor will be blind to treatment allocation. Placebo tablets will be matched to metformin tablets in size and appearance. Unblinding for all participating sites will be performed by the study sponsor whenever needed for safety reasons. For this purpose, a set of sealed envelopes comprising the information on the type of medication stored in every medication box used in the trial will be sent to the UMC Utrecht pharmacy. This pharmacy will provide a 24/7 backup emergency unblinding service. Participants will be excluded from subsequent statistical analyses after unblinding of the treatment allocation.

### Trial design

The trial will take place in a 52-week timeframe, including 26 weeks of treatment in both randomization groups. Metformin will be started at 500 mg b.i.d. (oral) and escalated to 1000 mg b.i.d. (4 tablets of 500 mg (=2000 mg)) 2 weeks thereafter. For participants aged 16–18 years, metformin dosage will be escalated to 500 mg b.i.d*. *maximum. Participants will undergo 4 main face-to-face visits after 0, 13, 26 and 52 weeks, as well as one short telephone visit after 2 weeks to evaluate the study medication dose (Table 1). Meanwhile, participants will undergo a lifestyle intervention that is considered care as usual in the participating centers. The study medication will start in the same seven days as the lifestyle intervention. The lifestyle intervention will exist of a combination of an exercise program and dietary interventions. The dietary intervention consists of five consultations with a dietitian in the first 26 weeks of treatment to stimulate both healthy food and appropriate caloric intake. The exercise program exists of minimally 60 min per week of unsupervised exercise by choice (i.e. walking, dancing, cycling or jogging). Also, participants gather in weekly lifestyle group sessions coached by an exercise professional with affinity in mental healthcare for low-intensity exercise including weekly weight measurements and assessment of physical activity using the Physical Activity Vital Sign questionnaire (PAVS) [40, 41]. Due to the COVID-19 pandemic, the weekly lifestyle sessions will be offered online. Participants may wish to discontinue lifestyle interventions at any moment and still continue with the current trial. Considering that some participants will discontinue lifestyle interventions in clinical practice, participants may carry on with the trial following the pragmatic design of this study to obtain results resembling clinical practice. Similarly, patients may continue with the trial after switching or stopping antipsychotic use. Patients should be on an antipsychotic at the start and may use any additional medicines except for medication(s) that inhibit(s) metabolism of metformin (e.g. bictegravir, cobicistat, daclatasvir, dolutegravir and vandetanib) or otherwise interact(s) with metformin (in particular those with possible detrimental effects on kidney functioning, e.g. NSAIDs, ACE-inhibitors, diuretics and intravascular iodine). Therefore, participants using the aforementioned medication will be excluded from this trial. Changes in medication use will be registered at each visit. Information regarding adherence to medication, dietary and exercise interventions is obtained by self-report during visits after 13 and 26 weeks. In addition, pill counts are used to assess medication use adherence. Participants are considered compliant in case of a pill count of > 80% [42, 43]. The adherence ratio will be calculated by the difference in the number of pills provided at visit 1 and the remaining pills at visit 3 and again between the number of pills provided at visit 3 and the remaining pills at visit 4 divided by the prescribed number of pills. This result will be multiplied by 100. To improve participant retention, participants will receive financial reimbursement for travel costs and 10 euros for each visit. Furthermore, the research team actively involved two experienced experts and patient association Anoiksis in the conceptualization and design of this study to ensure the design is patient-friendly, reflective of clinical practice and beneficial to patients.

**Table 1:**
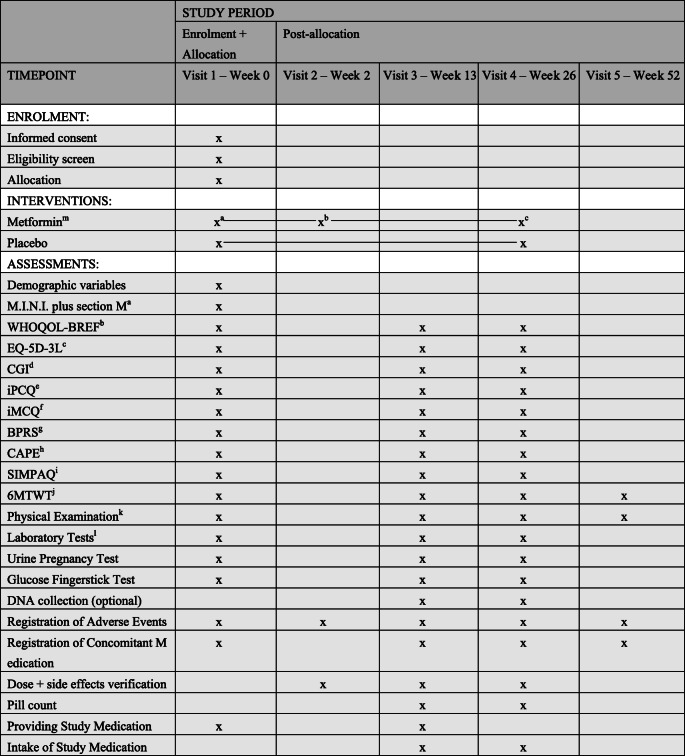
Study Flow Diagram

^a^Mini-International Neuropsychiatric Interview

^b^The short version of the official WHO quality of life questionnaire

^c^the 3-level EuroQol scale to describe and value health

^d^Clinical Global Impression

^e^iMTA Productivity Cost-effectiveness questionnaire

^f^iMTA Medical Consumption questionnaire

^g^Brief Psychiatric Rating Scale

^h^Community Assessment of Psychic Experiences

^i^Simple Physical Activity questionnaire

^j^6 Minutes Timed Walking Test

^k^Including height (first visit only), weight, BMI, systolic and diastolic blood pressure, pulse, waist circumference

^l^Including CRP, GFR, LDL, HDL, triglycerides, insulin, glucose, HbA1c

^m^x^a^ – x^b^ Metformin dose 1000 mg daily, x^b^ – x^c^ Metformin dose up to 2000 mg daily

### Assessments

#### Visit 1

During visit 1, baseline demographic characteristics will be assessed. Medical history including medication-, alcohol, tobacco- and substance use, weight before the start of mental illness, weight before the initiation of antipsychotic use and the estimated amount of weight gained due to antipsychotic use is also collected. The M.I.N.I. plus section M is used to assess the criteria for schizophrenia [44, 45]. All elements of MetS (i.e. body weight and height, BMI, waist circumference, and systolic and diastolic blood pressure) are measured during a short physical examination. The 6 Minutes Timed Walking Test (6MTWT) and Simple Physical Activity questionnaire (SIMPAQ) are used to assess physical endurance and physical activity, respectively [46]. Blood glucose levels are measured using a capillary blood fingerdipstick and urine pregnancy tests will be conducted for women of childbearing age. Additionally, blood will be drawn to measure C-reactive protein (CRP), GFR, Triglycerides, Low-density lipoprotein (LDL) and High-density lipoprotein (HDL) levels, insulin, glucose and Hemoglobin A1c (HbA1c). At last, several questionnaires will be assessed during visit 1 (see ‘secondary outcomes’ below).

#### Visit 2

Visit 2 is a short visit by telephone at 2 weeks into the study to assess how the participant is proceeding with the study medication, i.e. whether they have been able to switch from the starting dose of 500 mg b.i.d. to 1000 mg b.i.d. and whether they are experiencing any side effects.

#### Visits 3 and 4

During visits 3 and 4, adherence to lifestyle interventions is assessed, as well as information regarding medication use, dose and side effects, adverse events reactions, alcohol-, tobacco- and substance use. Similarly to visit 1, physical endurance, physical activity and elements of MetS are measured during physical examination additional to the blood glucose level using a capillary blood fingerdipstick. Also, a urine pregnancy test and laboratory tests will be conducted. One EDTA tube (6 cc) or saliva is optionally drawn for DNA extraction. Furthermore, the same questionnaires as during visit 1 will be taken.

#### Visit 5

One year after completing study visit 1, body weight, waist circumference and blood.

pressure are measured during physical examination and the 6MTWT is performed to assess physical endurance [46]. Furthermore, information regarding medication use and the (off-label) prescription of metformin between 26 weeks and 52 weeks by other physicians is assessed.

### Primary outcome

- Difference in body weight as a continuous trait between the two arms from treatment inception until 26 weeks of treatment.

### Secondary outcomes

Differences between the two arms from treatment inception until 26 weeks of treatment in:

- Any element of MetS (i.e., waist circumference (cm), BMI (kg/m^2^), triglycerides (mmol/L), LDL (mmol/L) and HDL (mmol/L) levels in blood, blood pressure (mmHg) and HbA1c (mmol/mol)) [3].
- Response, defined as ≥5% body weight loss at 26 weeks relative to treatment inception.
- Quality of life measured using the WHOQOL-BREF, a shorter version of the World Health Organization Quality of Life instrument [47].
- Quality of life measured using the complementary 3-level version of the EuroQol 5D (EQ-5D-3L) [48].
- General psychological and physical health using the brief Clinical Global Impression-Severity and -Improvent scale (CGI-S and CGI-I) [49].
- General psychological and physical health using the Brief Psychiatric Rating Scale (BPRS) [50].
- Potential fluctuations in depressive, positive and negative symptoms using the The Community Assessment of Psychic Experiences (CAPE) scale [51, 52].
- Cost-effectiveness using the iMTA Productivity Cost Questionnaire (iPCQ) and the iMTA Medical Consumption Questionnaire (iMCQ) [53].

### COVID-19 measures

During the face-to-face visits, measures will be taken in accordance with applicable guidelines regarding COVID-19 if necessary to optimize safety. Face-to-face visits can be adjusted as much as possible to online visits or house visits if required. The weekly lifestyle sessions will be offered online. Furthermore, the research team will use adequate protective equipment and will keep a physical distance of 1.5 m wherever possible. Additionally, participants will be asked to:

- keep a physical distance of 1.5 m from others
- avoid handshaking
- cough and sneeze in the elbow
- practice good hygiene (disinfect, wash hands)
- stay home if any cold or flu symptoms are present
- come to the face-to-face visits alone if possible

As in practice it may not be feasible for participants to attend the next visit exactly after 13 and 26 weeks, respectively, we allow for a 4-week time window for these visits, i.e. a participant should attend to the next visit within 11–15 weeks and 24–28 weeks, respectively. This is in line with clinical practice and the pragmatic nature of this trial: participants suffering from psychosis often miss follow-up consultations (due to hospitalization, negative symptoms or symptom exacerbation) and are then scheduled some weeks later. Furthermore, participants may be unable to attend due to COVID-19 symptoms. Participants with suspected or diagnosed COVID-19 may continue the trial whenever they are fully recovered within this time window.

### Data collection

Local authorized investigators will enter the acquired data and examination results into hard-copy documents and an electronic case record form (eCRF), Castor, that is accessible via the internet [54]**.** Investigators will receive personal user names and passwords for this purpose, and data will be encrypted for transfer. The investigator must (electronically) sign that entries into the eCRF are true and complete. After data has been submitted to the study centre, another thorough inspection of the completeness and plausibility of entries will be conducted. If needed, clarification questions will be addressed to the sites. Only after all questions regarding data quality have been answered, the database will be locked.

Pseudonymous (coded) data will be relayed to the central study team for scientific analysis or made available, if necessary, to the responsible supervisory authority. Only qualified and authorised collaborators of the study sponsor will enter the pseudonymous data into a computerised database. The acquired data will be used without participants’ names for scientific analysis and participants’ names will not be mentioned in any publication.

### Statistical analysis

#### Sample size calculation

A regression of weight on treatment with a sample size of 128 observations achieves 80% power at a two-sided 0.05 significance level to detect a difference in a weight reduction of two kgs (SD = 4, ES = 0.5) after 26 weeks (PASS 2008, version 8.0.16). We estimate the drop-out rate to be a maximum of 50% as such attrition rates are most commonly reported in trials targeting schizophrenia patients [37]. Drop-outs are those not attending the visit at 26 weeks. As soon as 128 subjects have completed this visit, inclusion will be stopped. Considering the estimated drop-out rate, we will therefore include approximately 256 subjects to ensure that 128 subjects complete the visit at 26 weeks. This power calculation is based on an independent two-sample t-tests of the outcome at 26 weeks. Using linear models in the data analysis will increase the efficiency of the analysis and therefore the power to detect a treatment effect.

#### Data analysis

The primary analysis in this trial tests the effect of metformin use next to care as usual including lifestyle interventions on body weight with a primary comparison at 26 weeks after treatment onset. This will be analyzed using a general linear mixed model with treatment group, baseline weight, gender, age, timepoint (as categorical factor) and compliance with lifestyle therapy as fixed factors and patient as random factors. Measurements of visit 3 (week 13) and visit 4 (week 26) will be included in the primary analysis models. Missing baseline parameters will be imputed before the analyses, but mixed models can handle missing outcome variables at post-randomization weeks if these are missing at random (MAR). The pattern of missingness will be examined. As a sensitivity analysis, body weight at 26 weeks will also be analyzed using an ANCOVA with treatment group and gender as factors and baseline weight and age as covariates. The significance threshold is set at *p* < 0.05. Considering the study population, we expect a high number of dropouts [37]. Additionally, we expect participants within one stratification group may switch clozapine or other antipsychotic use. Taking into account the pragmatic design of this study, the Intention-To-Treat principle will be followed for the primary and secondary analyses. In secondary analysis, the effect of metformin on body weight after 26 weeks of treatment will be compared to body weight at 52 weeks after 26 weeks of non-treatment with metformin. In this way, we can examine whether the possible effect of metformin on AiWG continues after 26 weeks of non-treatment following discontinuation of metformin use after 26 weeks of treatment. In a sensitivity analysis, per-protocol analysis will be applied for all analyses. Non-adherent participants to study medication defined as less than 80% of pills being taken will be excluded (see ‘Trial design’) [42, 43].

Similar analyses to the abovementioned primary study parameter analyses will be applied to the secondary outcomes, using generalized linear mixed models with appropriate link functions where necessary. For exploratory purposes, the following subgroup analyses within subtypes of patients will be conducted using the same model to explore possible differences in treatment effects: those on clozapine vs. those on other antipsychotics and those on high-risk agents (olanzapine, clozapine and quetiapine, risperidone and paliperidone) vs. all other antipsychotics [2, 12]. In post-hoc sensitivity analysis, the quantitative measure of the amount of self-reported AiWG will be used to examine differences between AiWG and weight gain due to other reasons.

For genetic analyses, we will run a linear model of polygenic risk scores (PRS) for BMI and MetS to assess their association with weight reduction. Covariates will be age, sex and three principal components of genetic ancestry. At each *p*-value threshold going from 5 × 10–8 to, 5 × 10–7, 5 × 10–6, 5 × 10–5, 5 × 10–4, 5 × 10–3, 0.01, 0.05, 0.1, 0.2, 0.3, and 0.4 both the variance in BMI/MetS and the p-value of the association will be computed to visualize the strength of association and degree of explained variance per p-value bin [55]. The optimal p-value threshold will be computed using PRSice (https://www.prsice.info/) or similar methods and reported. The significance threshold is set at *p* < 0.05.

### Monitoring

Monitoring will be performed by UMC Utrecht according to national laws and guidelines and the specifications of the ICH-GCP guidelines. Study monitors will visit study sites at regular intervals to monitor the execution of the study. Monitors will check whether requirements to conduct the study are met and study procedures are followed correctly, and will check the study site’s documentation, the participants’ source data, eCRF entries, and the correct maintenance of the Investigator Site File. The investigator will report all Serious Adverse Events (SAEs) to the sponsor through the eCRF without undue delay after obtaining knowledge of the events, with no exception.

## Discussion

Here, we describe the design of a pragmatic, randomized, double blind, multicenter controlled clinical trial to investigate whether metformin up to 2000 mg daily for 26 weeks in combination with treatment as usual including lifestyle interventions reduces AiWG in comparison to placebo in combination with lifestyle interventions.

The current trial has the following added value over earlier published trials regarding [29]. First, in line with clinical practice, where many patients treated with a range of antipsychotics report AiWG, inclusion is not restricted to certain antipsychotics, thus allowing us to evaluate whether this combination treatment is effective in the entire group of antipsychotics users. Second, the inclusion criteria are less strict than in previous trials, thus ensuring that the study population reflects clinical practice where some patients are not given a diagnosis of schizophrenia (but of another psychotic disorder) and may experience weight gain as problematic even if this is less than 10% (weight gain > 10% was an inclusion criterion in the Chinese 2008 trial) [28]. Third, the intervention is more feasible. The exercise program for participants in the current trial is less intense than other exercise programs: for example, our exercise program consists of two sessions weekly as opposed to seven in the published trial [28]. We expect that this increases feasibility and thus the likelihood of implementation in this patient group. Fourth, this is the first clinical trial examining the use of metformin in addition to lifestyle therapy for the treatment of AiWG in residents of a North-Western European country. This is relevant as cultural and genetic vulnerability factors may influence treatment adherence and efficacy. Fifth, we treat patients for 26 weeks as opposed to 12 weeks in the published trial, thus allowing us to better ascertain drop-outs and temporal patterns in treatment response [28]. At last, as it is currently impossible to predict who will benefit from treatment of AiWG with metformin we also include genetic analyses to assess whether polygenic risk scores are associated with metformin treatment response.

A unique element of this trial is the adaptation to the current COVID-19 pandemic by the possibility of online or house visits and online weekly lifestyle sessions. Furthermore, this study facilitates the evaluation of the efficacy of metformin in combination with lifestyle therapy on the elements of MetS, quality of life, general physical and mental health, physical endurance, symptoms of schizophrenia and depression and in relation to genetic liability. The pragmatic design of MELIA allows for a comparison of the efficacy and safety of a lifestyle intervention (i.e. exercise training and consultations with a dietician) with or without concomitant use of metformin in the treatment of AiWG. To conclude, we hope that this study will contribute to improved management strategies and new pharmacological guideline implementations for this major health problem.

## Data Availability

The dataset used and/ or analysed during the current study are available from the corresponding author on reasonable request.

## Abbreviations

ABR: General Assessment and Registration form (ABR form), the application form that is required for submission to the accredited Ethics Committee; in Dutch: Algemeen Beoordelings- en Registratieformulier (ABR-formulier)
AiWG: Antipsychotic induced Weight Gain
BMI: Body Mass Index
BPRS: Brief Psychiatric Rating Scale
CAPE: Community Assessment of Psychic Experiences
CGI: Clinical Global Impression
EQ-5D-3L: The three level EuroQol scale to describe and value health
GCP: Good Clinical Practice
GLP-1: Glucagon-Like-Peptide 1
GP: General Practitioner
iMCQ: iMedical Consumption Cost Questionnaire
iPCQ: iProductiivity Cost Questionnaire
METC: Medical research ethics committee (MREC); in Dutch: medisch-ethische toetsingscommissie (METC)
MetS: Metabolic Syndrome
M.I.N.I: Mini-International Neuropsychiatric Interview
NTR: Netherlands Trial Register
PAVS: Physical Activity Vital Sign questionnaire
PRS: Polygenic Risk Score
(S)AE: (Serious) Adverse Event
SIMPAQ: Simple Physical Activity Questionnaire
Sponsor: The sponsor is the party that commissions the organisation or performance of the research, for example a pharmaceutical company, academic hospital, scientific organisation or investigator. A party that provides funding for a study but does not commission it is not regarded as the sponsor, but referred to as a subsidising party
UMCU: University Medical Center Utrecht
WHOQOL-BREF: The short version of the official WHO quality of life questionnaire
WMO: Medical Research Involving Human Subjects Act; in Dutch: Wet Medisch-wetenschappelijk Onderzoek met Mensen
6MTWT: 6 Minutes Timed Walking Test
